# Optimizing implementations of linear layers using two and higher input XOR gates

**DOI:** 10.7717/peerj-cs.1820

**Published:** 2024-01-19

**Authors:** Meltem Kurt Pehlivanoğlu, Mehmet Ali Demir

**Affiliations:** Department of Computer Engineering, Kocaeli University, Kocaeli, Turkey

**Keywords:** Lightweight cryptography, Straight-line programs, Linear layers, MDS matrix, Low area circuits

## Abstract

Maximum distance separable (MDS) matrices are often used in the linear layer of a block cipher due to their good diffusion property. A well-designed lightweight MDS matrix, especially an involutory one, can provide both security and performance benefits to the cipher. Finding the corresponding effective linear straight-line program (SLP) of the circuit of a linear layer is still a challenging problem. In this article, first, we propose a new heuristic algorithm called Superior Boyar-Peralta (SBP) in the computation of the minimum number of two-input Exclusive-OR (XOR) gates with the minimum circuit depth for the SLPs. Contrary to the existing global optimization methods supporting only two-input XOR gates, SBP heuristic algorithm provides the best global optimization solutions, especially for extracting low-latency circuits. Moreover, we give a new 4 × 4 involutory MDS matrix over F_2^4^_, which requires only 41 XOR gates and depth 3 after applying SBP heuristic, whereas the previously best-known cost is 45 XOR gates with the same depth. In the second part of the article, for further optimization of the circuit area of linear layers with multiple-input XOR gates, we enhance the recently proposed BDKCI heuristic algorithm by incorporating circuit depth awareness, which limits the depth of the circuits created. By using the proposed circuit depth-bounded version of BDKCI, we present better circuit implementations of linear layers of block ciphers than those given in the literature. For instance, the given circuit for the AES MixColumn matrix only requires 44 XOR gates/depth 3/240.95 GE in the STM 130 nm (simply called ASIC4) library, while the previous best-known result is 55 XOR gates/depth 5/243.00 GE. Much better, our new 4 × 4 involutory MDS matrix requires only 19 XOR gates/depth3/79.75 GE in the STM 90 nm (simply called ASIC1) library, which is the lightest and superior to the state-of-the-art results.

## Introduction

In recent years, lightweight cryptography has gained increasing attention due to the usage of resource-constrained devices like smart devices, wearable devices, and Internet of Things (IoT) devices. Since these small, constrained devices can manipulate private data, designing novel, lighter cryptographic primitives with low implementation costs is crucial (Duval & Leurent, 2018).

Basically, a block cipher includes three components: a non-linear layer, a linear (or diffusion) layer, and a key schedule. A diffusion layer with a maximum branch number of $n + 1$, that uses a diffusion matrix of size $n \times n$ facilitates the measurement of diffusion rate in block ciphers. This diffusion layer ensures maximum diffusion, and matrices that satisfy this condition, such as MDS matrices, are commonly utilized in block ciphers (Rijmen et al., 1996; Daemen, Knudsen & Rijmen, 1997; Daemen & Rijmen, 2002; Schneier et al., 1998). Furthermore, MDS matrices are used in stream ciphers (Watanabe et al., 2002) and hash functions (Barreto, Rijmen & Nv, 2000; Choy et al., 2012; Gazzoni Filho, Barreto & Rijmen, 2006; Gauravaram et al., 2009; Guo, Peyrin & Poschmann, 2011), as indicated in various studies. In the literature, there are different types of methods to construct efficient MDS matrices, such as direct construction (Cui, Jin & Kong, 2015; Sajadieh et al., 2012; Gupta & Ray, 2013; Güzel et al., 2019), search based construction (Wu, Wang & Wu, 2013; Chand Gupta & Ghosh Ray, 2014; Li & Wang, 2016; Sarkar & Syed, 2016, 2017; Sakalli et al., 2020), and hybrid construction (Pehlivanoğlu et al., 2018).

Despite having maximum diffusion property, MDS matrices have high implementation costs. So, finding lightweight MDS diffusion layers, especially involutory ones, with minimized hardware requirements is a challenging task (Toh et al., 2018). The cost of implementation can be quantified using two key metrics: XOR count (which is divided into three types: direct-XOR (d-XOR), general-XOR (g-XOR), and sequential XOR (s-XOR) count (see “Definition and Notations” section for more details)) and circuit depth. XOR count represents the number of XOR gates required for the circuit implementation of the diffusion matrix, while circuit depth refers to the number of layers of gates required to implement the linear layer in hardware. Minimizing the number of gates, particularly expensive ones such as XOR gates ensures low-cost and efficient circuit implementations of the diffusion matrix. Similarly, minimizing circuit depth guarantees reduced latency. On the other hand, Gate Equivalent (GE) is a metric utilized to compare the size of logic gates. Banik, Funabiki & Isobe (2019) observed that utilization of higher input XOR gates may result in a reduced area (*i.e*., GE) in ASIC libraries. That idea brought new directions in research on optimized circuit implementations; hence, the researchers started to search for circuits not only with the minimum number of XOR gates and circuit depth but also with reduced GE cost by considering multiple-input XOR gates (*e.g*., Baksi et al., 2021; Banik, Funabiki & Isobe, 2019; Liu et al., 2022b).

### Related work

To address the challenge of finding efficient circuit implementations of a given linear layer, in the beginning, a variety of local optimization techniques (*e.g*., Gupta & Ray, 2013; Sim et al., 2015; Beierle, Kranz & Leander, 2016; Sarkar & Sim, 2016; Sarkar & Syed, 2017; Pehlivanoğlu et al., 2018) were proposed in order to reduce the number of XOR counts. Local optimization means the selection of the coefficients of the matrix with minimum XOR counts, but it does not guarantee the finding of efficient circuits. Because the fixed cost of connecting entries remains without being optimized in local optimization methods.

Then, the authors started to address the task of globally optimizing linear layers. This involves estimating the hardware cost of a linear layer by identifying an SLP that corresponds to it (Li et al., 2019). Two such algorithms are the Paar1 and Paar2 heuristics (Paar, 1997), which generate cancellation-free SLPs that do not contain the same variables in both variables of an XOR pair. Although Paar’s heuristics do not necessarily yield the optimal circuit implementations, the Paar1 heuristic is easy to implement and produces fast results, even for large matrix sizes. Boyar-Peralta’s heuristic (Boyar & Peralta, 2010) and its variant (Boyar, Matthews & Peralta, 2012) were an inspiration to improve new global optimization heuristics. In Boyar, Find & Peralta (2017), the authors modified Paar’s heuristic by using preprocessing steps and allowing cancellations. In the same article, minimizing the number of AND gates was handled beside XOR gates. In Duval & Leurent (2018), the authors proposed a new approach based on searching the circuit space to find optimal circuits of MDS matrices by using the tree-based Dijkstra searching technique. In Li et al. (2019), the authors modified Boyar-Peralta’s heuristic (Boyar, Matthews & Peralta, 2012) by considering the circuit depth metric to determine the optimal circuit implementations. Boyar, Find & Peralta (2019) proposed a new heuristic creating smaller linear and nonlinear circuits for a given circuit depth bound. Tan & Peyrin (2019) proposed Randomized Normal Boyar Peralta (RNBP) heuristic and two non-deterministic algorithms A1 and A2. All these given heuristics focus on the reduction of XOR counts by using temporary intermediate signals (gates) to determine the globally optimized implementations of a diffusion matrix. In Banik, Funabiki & Isobe (2021), the authors extracted lower circuit depth implementations by adding randomness in the tweaked algorithm given in Li et al. (2019) (this new version is simply called BFI heuristic). In Liu et al. (2022a), especially considering the low latency criteria, the authors proposed a new framework based on forward and backward search strategies that can find optimal solutions with a minimized circuit depth. Pehlivanoglu & Demir (2022) designed a new framework that combines some of these recently proposed global optimization heuristics to find better circuit implementations. It should be noted that all these global optimization methods given above generate circuit implementations with two-input XOR gates under the g-XOR metric.

The idea given in Banik, Funabiki & Isobe (2019) has opened up new directions for research on the usage of multiple-input XOR gates in SLP. In the same article, Banik, Funabiki & Isobe (2019) designed a graph-based heuristic to explore circuits featuring both two-input and three-input XOR gates. Specifically, they converted circuits constructed using only two-input gates into new ones with a combination of two-input and three-input XOR gates. Then, Baksi et al. (2021) introduced enhanced versions of BP heuristic (originally presented in Boyar & Peralta (2010) and Tan & Peyrin (2019)), simply called BDKCI. These improved versions support two-input, three-input, and four-input XOR gates. Recently, Liu et al. (2022b) proposed two algorithms: the transform algorithm and the graph extending algorithm. By combining these two algorithms, they generated better circuit implementations.

In the literature, there are few heuristics based on optimizing implementations of diffusion matrices under only the s-XOR metric without using any temporary intermediate signals. Optimizing a diffusion matrix under the s-XOR metric is based on the problem of optimal pivoting in the Gauss-Jordan elimination (Kölsch, 2019). In Jean et al. (2017), the authors proposed an exhaustive search algorithm to find out the optimal circuit implementations for small matrix sizes such as $4 \times 4$ and $8 \times 8$ under s-XOR metric. Xiang et al. (2020) proposed a new heuristic, called XZLBZ, that was capable of reducing the implementation cost (in terms of s-XOR count) of $16 \times 16$ and $32 \times 32$ involutory/non-involutory binary MDS matrices. More recently, Yang, Zeng & Wang (2021) proposed a new heuristic, called IX algorithm, which was an improved variant of the heuristic given in Xiang et al. (2020). IX heuristic found better circuit implementation under the same run-time with higher accuracy. However, the circuit depth metric is not taken into consideration in all of these heuristics designed to decrease the s-XOR count.

### Motivation and our contribution

In this article, we focus on two challenging research questions: (1) how to improve BP heuristic by considering the circuits using two-input XOR gates with low latency criteria (especially for depth 3), and (2) how to enhance BDKCI heuristic by incorporating circuit depth awareness. To address the first research question, we propose a new heuristic, called SBP, that is the improved version of Boyar-Peralta’s heuristic (Boyar, Matthews & Peralta, 2012) by considering low latency criteria. We introduce a new randomized way of choosing actions that would lead to better circuit solutions, especially with minimum circuit depth 3. To address the second research question, we give the enhanced (depth-bounded) version of BDKCI heuristic that is capable of producing depth-limited circuits.

The main contributions of this article can be given as follows:

- We give a new $4 \times 4$ involutory MDS linear layer over ${{\bf{F}}_{{2^4}}}$ whose circuit implementation requires the lowest number of XORs (*i.e*., 41 g-XORs saving four from the previous best result (Liu et al., 2022a)) with the minimum depth 3.
- We apply our new heuristic SBP to the previously given $4 \times 4$ linear layers and find many low-latency circuits which are better than the other best previous results given in Liu et al. (2022a).
- For further improvement, we enhance the recently proposed BDKCI heuristic algorithm by incorporating circuit depth awareness, which limits the depth of the circuits created.
- By using the proposed circuit depth-bounded version of BDKCI, we present better circuit implementations of linear layers of block ciphers than those given in the literature. Moreover, the given circuit for the AES MixColumn matrix only requires 44 XOR gates/depth 3/240.95 GE in ASIC4 library, while the previous best-known result is 55 XOR gates/depth 5/243.00 GE.
- Our new $4 \times 4$ involutory MDS matrix requires only 19 XOR gates with depth 3 by using the circuit depth-bounded version of BDKCI. In the ASIC1, ASIC2 (STM 65 nm), ASIC3 (TSMC 65 nm), and ASIC4 libraries, the circuit costs are 79.75, 88.486, 100.65, and 101.84 GE, respectively. These results not only outperform the state-of-the-art but also demonstrate that our circuit has the lowest cost.

All the source codes (SBP heuristic, depth-bounded version of BDKCI) and experimental results are available at https://github.com/demirmehmet0/SBP.

### Organization

This article is organized as follows: In “Definition and Notations” section, we give some basic notations and definitions. In “SBP Heuristic” section, we propose a new heuristic SBP for global optimization to generate low-latency circuit implementations of linear layers. Next, in “Depth-bounded version of BDKCI Heuristic” section, we present the depth-bounded version of BDKCI heuristic and some good experimental results. Finally, we conclude and highlight some possible future works for further results in “Conclusion and Future Works” section.

## Definition and notations

This section reviews the fundamental mathematical principles concerning finite fields and MDS matrices. In this context, definitions and notations are introduced.

The finite field ${{\bf{F}}_{{2^m}}}$ is defined by an irreducible polynomial $p(x)$ of degree $m$ over ${{\bf{F}}_2}$, can be denoted as ${{\bf{F}}_2}[x]/(p(x))$. Each element in finite field ${{\bf{F}}_{{2^m}}}$ can be represented as $\sum\nolimits_{i = 0}^{m - 1} {{b_i}} {\alpha ^i}$, where ${b_i}$ $\in$ ${{\bf{F}}_2}$ and $\alpha$ is a root of ${{\bf{F}}_{{2^m}}}$. For simplicity’s sake, the hexadecimal notation is used to represent the elements of ${{\bf{F}}_{{2^m}}}$ and the irreducible polynomial $p(x)$, *e.g*., the irreducible polynomial $p(x) = {x^4} + x + 1$ can be denoted as 0x13.

The $n \times n$ matrix over finite field ${{\bf{F}}_{{2^m}}}$ can be represented as ${M_n}({{\bf{F}}_{{2^m}}})$, and binary representation of the same $n \times n$ matrix (whose each entry is $m \times m$ invertible binary matrix) over the same finite field can be denoted as ${M_n}(GL(m,{{\bf{F}}_2}))$.

**Definition 1.** *(MDS Matrix) Let C be an* $[n,k,d]$ *code and* $G = [I|A]$ *be a generator matrix of C, where A is a* $k \times (n - k)$ *matrix. If and only if every square sub-matrix of A is nonsingular, A is an MDS matrix. If A also satisfies* $A = {A^{ - 1}}$, *A is an involutory MDS matrix*.

GHadamard matrix form proposed in Pehlivanoğlu et al. (2018) is a hybrid construction method to construct (involutory) MDS matrices. A $k \times k$ GHadamard matrix $GH$ is generated by using the non-zero ${b_i}$ parameters and their inverses with a $k \times k$ Finite Field Hadamard (simply Hadamard) matrix $H$ over ${{\bf{F}}_{{2^m}}}$. A $4 \times 4$ GHadamard matrix $GH$ can be denoted as follows:

**Definition 2.** *(GHadamard Matrix) Let* $H = \left [{\matrix{{a_0} & {a_1} & {a_2} & {a_3}\\ {a_1} & {a_0} & {a_3} & {a_2} \\ {a_2} & {a_3} & {a_0} & {a_1} \\ {a_3} & {a_2} & {a_1} & {a_0} } } \right]$ *be a* $4 \times 4$ *Hadamard* *matrix, and* $4 \times 4$ *GHadamard matrix form* $GH = Ghad({a_0},{a_1};{b_1},{a_2};{b_2},{a_3};{b_3})$, *where* ${b_1},{b_2}$ *and* ${b_3} \in {{\mathbb F}_{{2^m}}} - \{ 0\}$ *can be shown as follows:*

(1) $$GH = \left[ {\matrix{ {{a_0}} \hfill & {{a_1}{b_1}} \hfill & {{a_2}{b_2}} \hfill & {{a_3}{b_3}} \hfill \cr  {{a_1}b_1^{ - 1}} \hfill & {{a_0}} \hfill & {{a_3}b_1^{ - 1}{b_2}} \hfill & {{a_2}b_1^{ - 1}{b_3}} \hfill \cr  {{a_2}b_2^{ - 1}} \hfill & {{a_3}b_2^{ - 1}{b_1}} \hfill & {{a_0}} \hfill & {{a_1}b_2^{ - 1}{b_3}} \hfill \cr  {{a_3}b_3^{ - 1}} \hfill & {{a_2}b_3^{ - 1}{b_1}} \hfill & {{a_1}b_3^{ - 1}{b_2}} \hfill & {{a_0}} \hfill \cr  } } \right]$$

### Metrics

To compute the hardware implementation cost of a diffusion matrix in terms of XOR count, there are three important metrics: direct XOR (d-XOR) count (Khoo et al., 2014), sequential XOR (s-XOR) count (Jean et al., 2017), and general-XOR (g-XOR) count (we used the same abbreviation given in Xiang et al. (2020)).

**Definition 3.** *The d-XOR count is defined as the Hamming weight (sum of the nonzero elements) of the* $n \times n$ *invertible binary matrix minus* $n$.

**Definition 4.** *The s-XOR count is defined as the minimum number of XOR operations necessary to implement an* $n \times n$ *invertible binary matrix using in-place operations. In other words, given input vectors* $\{ {x_0},{x_1}, \ldots ,{x_{n - 1}}\}$ *of the* $n \times n$ *invertible binary matrix, the output vectors* $\{ {y_0},{y_1}, \ldots ,{y_{n - 1}}\}$ *are calculated using in-place operations* ${x_i} \leftarrow {x_i} \oplus {x_j}$, *where* $0 \le i,j \le n - 1$.

**Definition 5.** *The g-XOR is defined as the minimum number of required operations* ${x_i} \leftarrow {x_{{j_1}}} \oplus {x_{{j_2}}}$, *where* $0 \le {j_1},{j_2} \le i$.

Some intermediate values can be computed repeatedly under d-XOR and that will ensure a more costly (*i.e*., overestimation) final circuit than the actual one (Yang, Zeng & Wang, 2021), therefore s-XOR and g-XOR metrics are used for further evaluation. However, s-XOR count causes a high computational cost, especially for optimizing full MDS matrices (Duval & Leurent, 2018).

## Sbp heuristic

SBP heuristic starts from Boyar-Peralta’s heuristic but uses a different structure to find the optimal circuit solutions while choosing the new bases. SBP chooses a threshold value that gives the number of pair candidates that ensure (minimize the sum of distances or maximize the Euclidean Norm) the best results above the tie. After that, it performs a randomization step to randomly pick one of the best pairs by using the uniform integer distribution function. This function produces integer values in a range [0, threshold value] according to a uniform discrete distribution. Different distributions like uniform, normal, and sampling distributions were tested in our initial experiments exploring the effects of various random number distributions based on the Mersenne Twister algorithm (Matsumoto & Nishimura, 1998). The findings showed that the uniform integer distribution was the most effective (in terms of the XOR count of the generated circuit) in our research problem. Therefore, we chose it for further experiments.

We present all the details in Algorithm 1. According to Algorithm 1, *S* denotes a sequence of input signals (*i.e*., ${x_i}$s), *D* keeps trace of circuit depth of *S*, and $\Delta$ defines a distance vector, where ${\delta _H}(S,{y_i}s)$ represents the Hamming-Distance from *S* to output signals (*i.e*., ${y_i}$s). SBP picks signal pairs that maximize the Euclidean norm of the new updated distance vector $\Delta$, by taking into account the circuit depth limit. But here, the algorithm handles a specified number of pairs (depending on the $chosenParam$ parameter). Then, SBP applies the uniform discrete distribution function to determine a new base element. It performs the previous steps until all elements of $\Delta$ are equal to zero. The idea given in SBP potentially leads to the best result by pairing up the input signals that minimize the target values in the distance vector. The $chosenParam$ parameter plays a pivotal role in defining the dimension of the element space. If this space’s size equals the maximum count of selectable elements, SBP will yield outcomes equal to those of other optimization algorithms. However, by constraining the number of elements within this space (by using the $chosenParam$ parameter value), SBP consistently selects superior elements. When determining the $chosenParam$ value, it can be selected based on: (1) the size of the matrix, and (2) the runtime of other optimization algorithms. For small matrix sizes, the $chosenParam$ value should be lower compared to larger sizes. For the second condition, essentially, if generating the circuit for the same matrix takes a long time in other optimization algorithms, it is advisable to keep the $chosenParam$ value low, and if it takes a short time, a higher threshold is recommended. However, when the $chosenParam$ value is set excessively high, the algorithm may enter an infinite loop, making it challenging to make selections between elements or find any optimal element at all. When establishing the maximum value for the threshold, it is important to consider the fundamental factor, which is the number of elements the algorithm places in the candidate list (*i.e*., $allElement$ array) during each element selection. For example, if there are 10 elements in the $allElement$ array within one iteration, the threshold value should not exceed 10. However, since this situation varies with each iteration of the algorithm, a precise threshold value calculation cannot be made. Therefore, an average threshold value can be determined instead.

**Algorithm 1:** SBP algorithm.

1: **Input:** (*depthLimit, n, m, M*) $\triangleright$ /* *M*: a (*n×m*) binary matrix */
2: **Output:** *S* $\triangleright$ /* that evaluates optimized decomposition of *M* */
3: **Initialization**
4: $S \leftarrow [{x_1},{x_2},...,{x_n}]$ $\triangleright$ /* The input signals */
5: $D \leftarrow [0,0,...,0]$ $\triangleright$ /* keeps trace of the circuit depth of S, it is initialized to zero */
6: $\Delta\leftarrow[\delta_H(S,y_1),\ldots,\delta_H(S,y_m)]$ $\triangleright$ /* The distances and the initial distance equals Hamming Weight of the row minus one */
7: $j \leftarrow n$
8: **while** $\Delta \not = 0$ **do**
9: $besti \leftarrow 0$
10: $bestj \leftarrow 0$
11: $bestDist \leftarrow [0,0,...,0]$
12: $counter \leftarrow 0$
13: **for** $i \leftarrow$ 0 to *BaseSize* **do**
14: **if** $depth[i] + 1 \ge depthLimit$ **then**
15: continue
16: **end if**
17: **for** $j \leftarrow$ i+1 to *BaseSize* **do**
18: **if** $depth[j] + 1 \ge depthLimit$ **then**
19: continue
20: **end if**
21: $depthNewBase \leftarrow pow(2,Max(i,j) + 1)$
22: $thisDist \leftarrow totalDistance()$
23: **if** $thisDist \le minDistance$ **then**
24: $thisNorm \leftarrow Update$(thisNorm)
25: **if** $thisDist\lt minDistance$ **then**
26: **if** $depth[i] + 1 \ge depthLimit$ **then**
27: continue
28: **end if**
29: $thisNorm \leftarrow Update$(thisNorm)
30: **if** thisDist<minDistance || thisNorm*>*oldNorm **then**
31: **if** *counter > chosenParam* **then** $\triangleright$ /* *chosenParam*: defines the threshold value */
32: $counter \leftarrow 0$
33: **end if**
34: $allElement[counter] \leftarrow dist{\&}i{\&}j$
35: $counter + +$
36: **end if**
37: **end if**
38: **end if**
39: **end for**
40: **end for**
41: $number \leftarrow uniformIntDistribution(0,counter)$ $\triangleright$ /* Randomization */
42: $bestDist{\&}besti{\&}bestj \leftarrow allElement[number]$
43: *update*(*Base*) $\triangleright$ /* update base */
44: $update(D)$ $\triangleright$ /* update depth */
45: $update(\Delta )$ $\triangleright$ /* update distance */
46: **end while**
47: **return** *S*

While SBP shares its foundational traits with A1, A2, and RNBP, its superior performance can be attributed to its unique approach to element storage logic. Unlike other optimization algorithms that exhaustively explore all possibilities during element storage, thereby significantly expanding the search space and often generating numerous divergent paths, SBP takes a more controlled approach. SBP algorithm carefully curates the search space and stores elements acquired through the element selection process within BP algorithm, up to a specified limit. This strategy ensures that the highest-quality elements remain readily accessible within the stored values. The selections from this pool of top-tier elements facilitate a focus on achieving superior results. Consequently, this approach narrows down the search space, ultimately leading to the attainment of the optimal XOR count.

### Better circuit implementations for 4 × 4 low-latency involutory MDS matrices by using SBP heuristic

In this subsection, we apply our new heuristic SBP to the existing and new linear layers and find numerous low-latency candidates for circuit implementations. Notably, we give a new $4 \times 4$ involutory MDS matrix over ${{\bf{F}}_{{2^4}}}/$ 0x19 which can be implemented with only 41 g-XOR gates and depth 3 by applying SBP global optimization method, while the previous best optimal result requires 45 g-XOR gates (Liu et al., 2022a) for the same depth level.

**Example 6.** *Let* ${{\bf{F}}_{{2^4}}}$ *be generated by the primitive element* $\alpha$ *which is a root of the primitive polynomial* $0\times 19$. *Consider* $4 \times 4$ *Hadamard involutory MDS matrix* ${H_1} = had(1,{\alpha ^5},{\alpha ^{14}},{\alpha ^7})$ *over* ${{\bf{F}}_{{2^4}}}/$0x19. *Then, GHadamard matrix* $G{H_1} = Ghad(1,{\alpha ^5};{\alpha ^9},{\alpha ^{14}};{\alpha ^2},{\alpha ^7};{\alpha ^9})$ *corresponding to* ${H_1}$ *with parameters* ${b_1} = {\alpha ^9},{b_2} = {\alpha ^2}$, *and* ${b_3} = {\alpha ^9}$ *is given as follows:*

(2) $$G{H_1} = \left[ {\matrix{ 1 & {{\alpha ^{14}}} & \alpha & \alpha \cr  {{\alpha ^{11}}} & 1 & 1 & {{\alpha ^{14}}} \cr  {{\alpha ^{12}}} & {{\alpha ^{14}}} & 1 & {{\alpha ^{12}}} \cr  {{\alpha ^{13}}} & {{\alpha ^{14}}} & {{\alpha ^{13}}} & 1 \cr  } } \right]$$

*which is involutory and MDS matrix with d-XOR gate count* $69$ $( = 21 + 4 \times 3 \times 4)$. *After applying SBP heuristic to the matrix* $G{H_1}$, *we find the circuit with 41 g-XORs for depth 3*.

In Table 1, we provide the circuit implementation and computation sequence of the matrix $G{H_1}$ by applying SBP heuristic with threshold value 7. Moreover, we look for more efficient low latency circuit implementations of the matrix $G{H_1}$, so we compare our obtained implementation with the results from different state-of-the-art heuristics. We ran all the algorithms for eight hours for the matrix $G{H_1}$ by taking the number of XOR gates into account with respect to the minimum depth, then we present all the implementation costs in Table 2. As shown in Table 2, our proposed heuristic leads to better circuit results in terms of circuit depth (not only depth 3 but also different depths) than the other heuristics given in the literature.

**Table 1:** The global optimization result of ${{G}{H}_1}$ with 41 g-XORs and depth 3, where ${x_i}$ [ $({x_0},{x_1}, \ldots ,{x_{15}})$], ${y_j}$ [ $({y_0},{y_1}, \ldots ,{y_{15}})$] and ${t_k}$ [ $({t_1},{t_2}, \ldots ,{t_{41}})$] refer to the input signals, output signals, and temporary intermediate signals, respectively, and the values are given in parentheses refer to circuit depth.

Iter.	New base element	New distance vector $\Delta$
1	${t_1} = {x_1} + {x_9}$ (1)	$[3,3,4,5,4,3,6,4,5,4,6,3,3,5,5,2]$
2	${t_2} = {x_0} + {x_8}$ (1)	$[3,3,4,5,3,3,6,4,4,4,6,3,3,4,4,2]$
3	${t_3} = {x_2} + {x_{14}}$ (1)	$[3,3,4,5,3,2,6,4,4,4,5,2,3,4,4,2]$
4	${t_4} = {x_4} + {x_{13}}$ (1)	$[3,3,3,5,2,2,6,4,4,4,4,2,3,4,4,2]$
5	${t_5} = {x_0} + {x_{12}}$ (1)	$[3,3,3,5,2,2,5,3,4,3,4,2,3,4,4,2]$
6	${t_6} = {x_6} + {t_5}$ (2)	$[3,2,3,5,2,2,4,3,4,2,4,2,3,4,4,2]$
7	${t_7} = {x_1} + {t_2}$ (2)	$[3,1,3,5,1,2,4,3,4,2,4,2,3,4,4,2]$
8	${t_8} = {t_6} + {t_7}$ $[{y_1}]$ (3)	$[3,0,3,5,1,2,4,3,4,2,4,2,3,4,4,2]$
9	${t_9} = {t_4} + {t_7}$ $[{y_4}]$ (3)	$[3,0,3,5,0,2,4,3,4,2,4,2,3,4,4,2]$
10	${t_{10}} = {x_2} + {x_{10}}$ (1)	$[3,0,3,5,0,2,3,3,4,2,4,2,2,4,4,2]$
11	${t_{11}} = {x_3} + {x_{11}}$ (1)	$[3,0,3,4,0,2,3,2,4,2,4,2,2,3,4,2]$
12	${t_{12}} = {x_3} + {x_{15}}$ (1)	$[3,0,3,4,0,2,2,2,3,2,4,2,2,3,4,2]$
13	${t_{13}} = {x_4} + {x_{11}}$ (1)	$[3,0,3,3,0,2,2,2,3,2,4,1,2,3,4,2]$
14	${t_{14}} = {t_3} + {t_{13}}$ $[{y_{11}}]$ (2)	$[3,0,3,2,0,2,2,2,3,2,4,0,2,3,4,2]$
15	${t_{15}} = {x_5} + {x_{12}}$ (1)	$[3,0,3,2,0,2,2,2,2,2,4,0,1,3,4,2]$
16	${t_{16}} = {t_{10}} + {t_{15}}$ $[{y_{12}}]$ (2)	$[3,0,3,2,0,2,2,2,2,2,4,0,0,3,4,2]$
17	${t_{17}} = {t_5} + {t_{11}}$ (2)	$[2,0,3,2,0,2,2,1,2,2,4,0,0,3,4,2]$
18	${t_{18}} = {x_7} + {t_{17}}$ $[{y_7}]$ (3)	$[2,0,3,2,0,2,2,0,2,2,4,0,0,3,4,2]$
19	${t_{19}} = {x_4} + {t_1}$ (2)	$[2,0,3,2,0,2,2,0,2,2,4,0,0,3,3,1]$
20	${t_{20}} = {x_{15}} + {t_{19}}$ $[{y_{15}}]$ (3)	$[2,0,3,2,0,2,2,0,2,2,4,0,0,3,3,0]$
21	${t_{21}} = {x_7} + {x_{14}}$ (1)	$[2,0,3,2,0,2,2,0,2,2,3,0,0,3,2,0]$
22	${t_{22}} = {t_{10}} + {t_{12}}$ (2)	$[2,0,3,1,0,2,1,0,2,2,3,0,0,3,2,0]$
23	${t_{23}} = {t_{14}} + {t_{22}}$ $[{y_3}]$ (3)	$[2,0,3,0,0,2,1,0,2,2,3,0,0,3,2,0]$
24	${t_{24}} = {t_6} + {t_{22}}$ $[{y_6}]$ (3)	$[2,0,3,0,0,2,0,0,2,2,3,0,0,3,2,0]$
25	${t_{25}} = {t_{12}} + {t_{15}}$ (2)	$[1,0,3,0,0,2,0,0,1,2,3,0,0,3,2,0]$
26	${t_{26}} = {t_{17}} + {t_{25}}$ $[{y_0}]$ (3)	$[0,0,3,0,0,2,0,0,1,2,3,0,0,3,2,0]$
27	${t_{27}} = {t_2} + {t_{25}}$ $[{y_8}]$ (3)	$[0,0,3,0,0,2,0,0,0,2,3,0,0,3,2,0]$
28	${t_{28}} = {x_5} + {t_1}$ (2)	$[0,0,3,0,0,1,0,0,0,2,3,0,0,3,2,0]$
29	${t_{29}} = {t_3} + {t_{28}}$ $[{y_5}]$ (3)	$[0,0,3,0,0,0,0,0,0,2,3,0,0,3,2,0]$
30	${t_{30}} = {x_{13}} + {t_1}$ (2)	$[0,0,3,0,0,0,0,0,0,1,3,0,0,3,2,0]$
31	${t_{31}} = {t_6} + {t_{30}}$ $[{y_9}]$ (3)	$[0,0,3,0,0,0,0,0,0,0,3,0,0,3,2,0]$
32	${t_{32}} = {t_2} + {t_{21}}$ (2)	$[0,0,3,0,0,0,0,0,0,0,3,0,0,3,1,0]$
33	${t_{33}} = {t_{19}} + {t_{32}}$ $[{y_{14}}]$ (3)	$[0,0,3,0,0,0,0,0,0,0,3,0,0,3,0,0]$
34	${t_{34}} = {t_4} + {t_{21}}$ (2)	$[0,0,2,0,0,0,0,0,0,0,2,0,0,3,0,0]$
35	${t_{35}} = {x_1} + {t_{10}}$ (2)	$[0,0,2,0,0,0,0,0,0,0,1,0,0,3,0,0]$
36	${t_{36}} = {t_{34}} + {t_{35}}$ $[{y_{10}}]$ (3)	$[0,0,2,0,0,0,0,0,0,0,0,0,0,3,0,0]$
37	${t_{37}} = {x_9} + {t_3}$ (2)	$[0,0,1,0,0,0,0,0,0,0,0,0,0,3,0,0]$
38	${t_{38}} = {t_{34}} + {t_{37}}$ $[y2]$ (3)	$[0,0,0,0,0,0,0,0,0,0,0,0,0,3,0,0]$
39	${t_{39}} = {x_6} + {x_{13}}$ (1)	$[0,0,0,0,0,0,0,0,0,0,0,0,0,2,0,0]$
40	${t_{40}} = {t_2} + {t_{11}}$ (2)	$[0,0,0,0,0,0,0,0,0,0,0,0,0,1,0,0]$
41	${t_{41}} = {t_{39}} + {t_{40}}$ $[{y_{13}}]$ (3)	$[0,0,0,0,0,0,0,0,0,0,0,0,0,0,0,0]$

**Table 2:** Circuit cost (XOR count/depth) of $G{H_1}$ under several global optimization algorithms.

Matrix	Paar1 (Paar, 1997)	RPaar1 (Lin et al., 2021)	BP (Li et al., 2019)	A1 (Tan & Peyrin, 2019)	A2 (Tan & Peyrin, 2019)	RNBP (Tan & Peyrin, 2019)	Liu et al. (2022a)	SBP
$G{H_1}$	37/8^†^	37/5^†^	44/3*	41/5* (37/6^†^)	39/5* (37/5^†^)	41/4* (37/5^†^)	44/3*	(**41/3, 39/4**)

**Notes:**

* Extracted from its original algorithm.

† Extracted from the framework given in Lin et al. (2021).

Bold values indicate the best results.

Furthermore, in Table 3, we consider the several $4 \times 4$ linear layers given in the literature to extract their low latency circuits. Notably, our results are better than the other heuristics, we can easily see that the SBP heuristic ensures a significant improvement for the minimum circuit depth metric. Note that, the implementation of $4 \times 4$ involutory MDS matrix given in Sarkar & Syed (2016) requires only 44 g-XOR, and 40 g-XOR for depth 3 and depth 4, respectively. These new records beat all previous best-known results for this matrix. Even though we find a new record, the circuit of $G{H_1}$ (see Table 1) beats all the records (for low latency implementations of $4 \times 4$ involutory MDS linear layers).

**Table 3:** Comparison of circuit cost (XOR count/depth) of binary matrices of size 16 × 16 under several global optimization algorithms.

Matrix	Kranz et al. (2017)	Xiang et al. (2020)	Lin et al. (2021)	Li et al. (2019)	Banik, Funabiki & Isobe (2021)	Liu et al. (2022a)	SBP
SMALLSCALE AES (Cid, Murphy & Robshaw, 2005)	47/7	43/5	43/5	49/3	49/3	47/3	(48/3, 47/4)
JOLTIK (Jean, Nikolic & Peyrin, 2015)	48/4	44/7	43/8	51/3	50/3	48/3	49/3
MIDORI (Banik et al., 2015)	24/4	24/3	24/3	24/2	24/2	24/2	**24/2**
PRINCE ${M_0}$ (Borghoff et al., 2012)	24/4	24/6	24/6	24/2	24/2	24/2	**24/2**
PRINCE ${M_1}$ (Borghoff et al., 2012)	24/4	24/6	24/6	24/2	24/2	24/2	**24/2**
PRIDE ${L_0}$, ${L_3}$ (Albrecht et al., 2014)	24/4	24/3	24/3	24/2	24/2	24/2	**24/2**
PRIDE ${L_1}$, ${L_2}$ (Albrecht et al., 2014)	24/3	24/3	24/3	24/2	24/2	24/2	**24/2**
QARMA64 (Avanzi, 2017)	24/3	24/5	24/5	24/2	24/2	24/2	**24/2**
SKINNY (Beierle et al., 2016)	12/2	12/2	12/2	12/2	12/2	12/2	**12/2**
Non involutory MDS matrices							
Sim et al. (2015) (Hadamard)	48/3	44/7	44/7	51/3	50/3	49/3	**48/3**
Liu & Sim (2016) (Circulant)	44/3	44/6	43/4	47/3	44/3	44/3	47/3
Li & Wang (2016) (Circulant)	44/5	44/8	43/4	47/3	44/3	44/3	47/3
Beierle, Kranz & Leander (2016) (Circulant)	42/5	41/6	40/5	47/3	43/3	45/3	46/3
Sarkar & Syed (2016) (Toeplitz)	43/5	41/7	40/7	44/3	43/3	45/3	**43/3**
Jean et al. (2017)	43/5	41/6	40/6	45/3	45/3	45/3	(**45/3, 43/4**)
Involutory MDS matrices							
Sim et al. (2015)	48/4	44/8	43/8	51/3	49/3	48/3	(49/3, **47/4**)
Li & Wang (2016)	48/4	44/6	43/8	51/3	49/3	48/3	(49/3, **48/4**)
Sarkar & Syed (2016)	42/4	38/8	37/7	48/3	46/3	45/3	(**44/3, 40/4**)
Jean et al. (2017)	47/7	41/6	41/10	47/3	47/3	47/3	(**45/4**)

**Note:**

Bold values indicate the best results.

## Depth-bounded version of bdkci heuristic

BDKCI algorithm typically allows circuits to be generated without any limitations on circuit depth. However, in this article, we have improved upon this heuristic by introducing circuit awareness. We present algorithms just for the modified functions within the original BDKCI heuristic. Note that, we have not only made alterations to these two functions but have also modified others called within them.

Algorithm 2, represents the Main function that begins by importing a target matrix. It then systematically introduces XOR gates using the SLP method until all elements within the target matrix are encompassed. This iterative process is tailored to iteratively enhance the parameters of the XOR circuit through multiple applications of the SLP method. At the end of each iteration, the best XOR circuit parameters, including relevant information such as cost and depth, are recorded in a log file. On the other hand, Algorithm 3, represents the PickNewBaseElementXOR3 function. Basically, in this function, an element ( $chosen$ value) is chosen randomly from the element array to generate a circuit gate. Also, the depth value of the selected element is appended to the depth array. In the original BDKCI version, for detecting the $chosen$ value, A1, and A2 algorithms can be used in addition to RNBP. But, in our proposed depth-bounded version, we just utilize RNBP heuristic.

**Algorithm 2:** Main function.

1: $depths \leftarrow$ array of size 1,000
2: $DepthLimit \leftarrow 5$
3: **function** MAIN
4: ReadTargetMatrix $\triangleright$ /* Read TargetMatrix then construct Target, Dist arrays */
5: **while** iterations $\gt 0$ **do**
6: $BestCount,BestCost1,BestCost3,BestCost4,BestDepth \leftarrow$ LARGE
7: $XorCount,Xor2Count,Xor3Count,Xor4Count \leftarrow$ 0
8: $XorCost1,XorCost2,XorCost3,XorCost4 \leftarrow$ 0
9: refreshDistAndTarget $\triangleright$ /* Update the Target and Dist arrays by randomly shuffling TargetMatrix */
10: InitBase $\triangleright$ /* Set initial values */
11: $\_returnVal2 \leftarrow 0$ $\triangleright$ /* Set initial value for $\_returnVal2$ */
12: **while** TargetsFound < NumTargets **do**
13: $\_returnVal \leftarrow$ EasyMoveXOR3 $\triangleright$ /* Search for targets with a distance of 1 */
14: **if** $\_returnVal = 0$ **then**
15: PickNewBaseElementXOR3 $\triangleright$ /* Select new elements to create circuits with 3-input, and 4-input XOR gates */
16: **else if** $\_returnVal = 2$ **then**
17: $\_returnVal2 \leftarrow 2$
18: **break**
19: **end if**
20: **if** not EasyMove **then**
21: PickNewBaseElement $\triangleright$ /* Select new elements to create circuits with just 2-input XOR gates */
22: **end if**
23: **if** the difference between any BestCost and XorCost is greater than 0.001 **then**
24: **if** $\_returnVal2 \ne 2$ **then**
25: logs $\leftarrow$ trialNo
26: **end if**
27: $depth \leftarrow$ max_element(depth_map.begin(), depth_map.end())
28: **if** TargetsFound = NumTargets **then**
29: **if** IWSEC **then**
30: $\triangleright$ /* If all targets are found, the depth is calculated */
31: **end if**
32: **end if**
33: $t \leftarrow$ current time
34: **if** IWSEC **then**
35: **if** $\_returnVal \ne 2$ **then**
36: **end if**
37: **end if**
38: $\triangleright$ /* Based on the $\_returnVal2$ value, checks are made and results are written to the log. */
39: **end if**
40: logs.close()
41: **end while**
42: **end while**
43: **end function**

**Algorithm 3:** PickNewBaseElementXOR3 function.

1: **function** PickNewBaseElementXOR3
2: $AllElements \leftarrow$ allocate space for array of size $BaseSize \times (BaseSize - 1) \times (BaseSize \times BaseSize - 4 \times BaseSize + 5)$
3: $counter \leftarrow 0$
4: $DepthLimit \leftarrow chosencircuitdepthlimit$ $\triangleright$ /* Depending on the selected circuit depth limit, the *chosencircuitdepthlimit* value can be adjusted, *e.g*., 3,4, *etc*. */
5: **for** $i \in [0,BaseSize)$ **do**
6: **for** $j \in [i + 1,BaseSize)$ **do**
7: **if** $depths[i] + 1 \gt DepthLimit$ **or** $depths[j] + 1 \gt DepthLimit$ **then**
8: **continue**
9: **end if**
10: $NewBase \leftarrow Base[i] \oplus Base[j]$
11: TotalDistanceXOR3(Gate::XOR2) $\triangleright$ /* Store results of a 2-input XOR operation with distances and parent indices */
12: **for** $k \in [0,NumTargets)$ **do**
13: $AllElements[counter].newDist[k] \leftarrow NDist[k]$
14: **end for**
15: $AllElements[counter].paren{t_i} \leftarrow i$
16: $AllElements[counter].paren{t_j} \leftarrow j$
17: $AllElements[counter].gate \leftarrow Gate::XOR2$
18: $counter \leftarrow counter + 1$
19: **for** $k \in [j + 1,BaseSize)$ **do**
20: **if** $depths[i] + 1 \gt DepthLimit$ **or** $depths[j] + 1 \gt 4$ **or** $depths[k] + 1 \gt DepthLimit$ **then**
21: **continue**
22: **end if**
23: $NewBase \leftarrow Base[i] \oplus Base[j] \oplus Base[k]$
24: TotalDistanceXOR3(Gate::XOR3) $\triangleright$ /* Store results of a 3-input XOR operation with distances and parent indices */
25: **for** $l \in [0,NumTargets)$ **do**
26: $AllElements[counter].newDist[l] \leftarrow NDist[l]$
27: **end for**
28: $AllElements[counter].paren{t_i} \leftarrow i$
29: $AllElements[counter].paren{t_j} \leftarrow j$
30: $AllElements[counter].paren{t_k} \leftarrow k$
31: $AllElements[counter].gate \leftarrow Gate::XOR3$
32: $counter \leftarrow counter + 1$
33: **if** XOR4 is defined **then**
34: **for** $l \in [k + 1,BaseSize)$ **do**
35: $NewBase \leftarrow Base[i] \oplus Base[j] \oplus Base[k] \oplus Base[l]$
36: TotalDistanceXOR3(Gate::XOR4) $\triangleright$ /* Store results of a 4-input XOR operation with distances and parent indices */
37: **end for**
38: **end if**
39: **end for**
40: **end for**
41: **end for**
42: $chosen \leftarrow RNBP(AllElements,counter)$ $\triangleright$ The *chosen* variable holds a value returned from RNBP algorithm. RNBP algorithm selects one of the elements from the *AllElements* array, then returns its index in the array. This index is subsequently assigned to the “*chosen*” variable.
43: $\triangleright$ /* The remaining portion of the algorithm includes tasks such as updating the base, computing costs, and releasing memory resources. */
44: **end function**

The following is a brief overview of the changes made to the original BDKCI heuristic:

• Within the Main function, the “ $BestDepth$” variable is declared as a large data type, enabling it to store the minimum depth value identified during the algorithm’s execution. Inside the same function, we have established the “ $depths$” array for the purpose of retaining the depth of each gate. These values play a crucial role in identifying the minimum depth value attained throughout the algorithm’s execution.

• The return type of the EasyMoveXOR3 function has been altered to an integer, allowing us to decide whether to print the results based on the function’s return value. Moreover, inside the same function, we have made the following modifications that allow us to record depth information of two-input XOR gates, three-input XOR gates, and four-input XOR gates, respectively.

$depths[BaseSize] = max(depth\_map[a],depth\_map[b]) + 1$,

$ depths[BaseSize] = max(depth\_map[a],depth\_map[b],depth\_map[c]) + 1$, $\hskip0.4pc depths[BaseSize] = max(depth\_map[a],depth\_map[b],depth\_map[c],depth\_map[d]) + 1$.

Furthermore, within the EasyMoveXOR3 function, a boolean variable named “ $_foundone$” has been defined to monitor whether the algorithm’s depth surpasses the specified threshold value, thus influencing the progression or conclusion of the current algorithm round.

• In the function PickNewBaseElementXOR3, we have defined “ $DepthLimit$” variable that allows us to generate circuits with the chosen circuit depth. Moreover, the condition “ $if(depths[i] + 1 \gt DepthLimit||(depths[j] + 1 \gt DepthLimit)$” compares the depth information of the element pair that is eligible for selection in the current round with the depth limit. If the depth limit is exceeded, this pair of elements is not selected, and the loop continues to select a new pair of elements.

### Better circuit implementations by using depth-bounded version of BDKCI heuristic

In this subsection, we present improved circuit implementations for the linear layers of some block ciphers, utilizing the circuit depth-bounded version of the BDKCI heuristic suggested in this study. We enhanced AES MixColumn matrix circuit with a cost of 240.95 GE (see Table 4) for the ASIC4 library. This circuit utilizes five XOR2 gates, seven XOR3 gates, and 32 XOR4 gates with depth 3, outperforming the previous best result of 243 GE with depth 5 (Liu et al., 2022b). Note that, XOR2, XOR3, and XOR4 refer to two-input XOR gates, three-input XOR gates, and four-input XOR gates, respectively.

**Table 4:** The global optimization result of AES MixColumn matrix with 44 XORs, depth 3, and 240.95 GE in ASIC4 by using the depth-bounded version of BDKCI.

No	Operation	No	Operation
1	${t_0} = {x_{15}} \oplus {x_{23}}$	23	${y_{25}} = {x_{25}} \oplus {t_4} \oplus {t_8} \oplus {t_{20}}$
2	${y_{31}} = {x_6} \oplus {x_7} \oplus {x_{30}} \oplus {t_0}$	24	${y_2} = {x_2} \oplus {x_{18}} \oplus {y_{18}} \oplus {t_{20}}$
3	${y_7} = {x_6} \oplus {x_{14}} \oplus {x_{31}} \oplus {t_0}$	25	${t_{24}} = {x_{11}} \oplus {x_{20}} \oplus {x_{28}}$
4	${y_8} = {x_0} \oplus {x_{16}} \oplus {x_{24}} \oplus {t_0}$	26	${y_{12}} = {x_4} \oplus {x_{19}} \oplus {t_0} \oplus {t_{24}}$
5	${t_4} = {x_7} \oplus {x_{15}}$	27	${y_{04}} = {x_3} \oplus {x_{12}} \oplus {t_4} \oplus {t_{24}}$
6	${y_{15}} = {x_6} \oplus {x_{22}} \oplus {y_7} \oplus {t_4}$	28	${t_{27}} = {x_0} \oplus {x_8} \oplus {t_{20}}$
7	${y_0} = {x_8} \oplus {x_{16}} \oplus {x_{24}} \oplus {t_4}$	29	${y_{17}} = {x_{16}} \oplus {x_{17}} \oplus {y_{16}} \oplus {t_{27}}$
8	${y_{23}} = {x_{22}} \oplus {x_{31}} \oplus {x_{30}} \oplus {t_4}$	30	${y_1} = {x_1} \oplus {t_4} \oplus {t_{27}}$
9	${t_8} = {x_0} \oplus {x_{15}} \oplus {x_{24}} \oplus {x_{31}}$	31	${y_9} = {x_9} \oplus {x_{24}} \oplus {y_8} \oplus {t_{27}}$
10	${y_{24}} = {y_0} \oplus {t_8}$	32	${t_{31}} = {x_6} \oplus {x_{14}} \oplus {x_{29}}$
11	${y_{16}} = {x_8} \oplus {t_0} \oplus {t_8}$	33	${y_{22}} = {x_{21}} \oplus {x_{30}} \oplus {t_{31}}$
12	${t_{11}} = {x_3} \oplus {x_{11}} \oplus {x_{26}} \oplus {x_{31}}$	34	${y_{30}} = {x_5} \oplus {x_{22}} \oplus {t_{31}}$
13	${y_{27}} = {x_2} \oplus {x_7} \oplus {x_{19}} \oplus {t_{11}}$	35	${t_{34}} = {x_5} \oplus {x_{13}} \oplus {x_{21}} \oplus {x_{29}}$
14	${y_{19}} = {x_{18}} \oplus {x_{23}} \oplus {x_{27}} \oplus {t_{11}}$	36	${y_{14}} = {x_{14}} \oplus {x_{30}} \oplus {y_{30}} \oplus {t_{34}}$
15	${t_{14}} = {x_4} \oplus {x_{12}} \oplus {x_{27}} \oplus {x_{31}}$	37	${y_{21}} = {x_{11}} \oplus {x_{21}} \oplus {t_{24}} \oplus {t_{34}}$
16	${y_{20}} = {x_{19}} \oplus {x_{23}} \oplus {x_{28}} \oplus {t_{14}}$	38	${y_{13}} = {x_{13}} \oplus {x_{12}} \oplus {x_{20}} \oplus {t_{34}}$
17	${y_{28}} = {x_3} \oplus {x_7} \oplus {x_{20}} \oplus {t_{14}}$	39	${y_6} = {x_6} \oplus {x_{22}} \oplus {y_{22}} \oplus {t_{34}}$
18	${t_{17}} = {x_2} \oplus {x_{10}}$	40	${y_5} = {x_4} \oplus {x_5} \oplus {x_{12}} \oplus {t_{34}}$
19	${y_{26}} = {x_1} \oplus {x_{18}} \oplus {x_{25}} \oplus {t_{17}}$	41	${y_{29}} = {x_4} \oplus {x_{29}} \oplus {x_{28}} \oplus {t_{34}}$
20	${y_{18}} = {x_{17}} \oplus {x_{25}} \oplus {x_{26}} \oplus {t_{17}}$	42	${t_{41}} = {t_4} \oplus {t_{17}}$
21	${t_{20}} = {x_1} \oplus {x_9} \oplus {x_{17}} \oplus {x_{25}}$	43	${y_{11}} = {x_3} \oplus {y_{27}} \oplus {y_{19}} \oplus {t_{41}}$
22	${y_{10}} = {x_{10}} \oplus {x_{26}} \oplus {y_{26}} \oplus {t_{20}}$	44	${y_3} = {x_{11}} \oplus {x_{27}} \oplus {x_{19}} \oplus {t_{41}}$

The binary matrix of AES MixColumn is directly taken from the repository given in Baksi et al. (2021). Table 5 provides an overview of recent works that have utilized AES MixColumn, including our own findings. Additionally, we have enhanced the previous implementations of linear layers for ANUBIS and CLEFIA ${M_0}$. As for TWOFISH, we find the circuit which equals the previous best-known result. Table 6 contains the comparison results for these various diffusion layers. Moreover, for further optimization, we globally optimized $G{H_1}$ by using the depth-bounded version of BDKCI. The optimized circuit implementation of $G{H_1}$ is given in Table 7. It requires only one XOR2 gate, seven XOR3 gates, and 11 XOR4 gates with depth 3. Additionally, we have compared our result with those of other $4 \times 4$ involutory and MDS matrices over ${{\bf{F}}_{{2^4}}}$ for ASIC1, ASIC2, ASIC3, and ASIC4 libraries. The results presented in Table 8 indicate that our matrix has the smallest GE values for all ASIC libraries.

**Table 5:** A brief overview of recent implementation costs of the AES MixColumn matrix in ASIC4.

Ref.	#XOR2	#XOR3	#XOR4	GC	Depth	GE
Banik, Funabiki & Isobe (2019)	95	–	–	95	6	316.35
Banik, Funabiki & Isobe (2019)	39	28	–	67	6	260.35
Tan & Peyrin (2019)	94	–	–	94	9	313.02
Maximov (2019)	92	–	–	92	6	306.36
Xiang et al. (2020)	92	–	–	92	6	306.36
Lin et al. (2021)	91	–	–	91	7	303.03
Baksi et al. (2021)	12	47	–	59	4	258.98
Liu et al. (2022b)	22	21	12	55	5	243.0
This article	5	7	32	**44**	**3**	**240.95**

**Note:**

The notations “#XOR2, #XOR3, #XOR4” indicate the number of two-input XOR gates, three-input XOR gates, and four-input XOR gates needed, respectively. Bold values indicate the best results.

**Table 6:** Summary of implementation costs of linear layers of various block ciphers in ASIC4 library.

Matrix	XZLBZ (Xiang et al., 2020)	BDKCI (Baksi et al., 2021)	BFI (Banik, Funabiki & Isobe, 2021)	XZLBZ+BFI (Liu et al., 2022b)	XZLBZ+EGT2 (Liu et al., 2022b)	XZLBZ+EGT3 (Liu et al., 2022b)	This article
ANUBIS (Barreto & Rijmen, 2000)	329.6	274.2	293.0	270.3	270.3	253.6	**251.61**
CLEFIA ${M_0}$ (Shirai et al., 2007)	326.3	271.63	293.0	276.3	270.9	258.9	**256.27**
CLEFIA ${M_1}$ (Shirai et al., 2007)	342.9	298.9	294.3	292.9	283.6	**270.2**	286.88
JOLTIK	146.5	122.5	127.8	126.5	123.8	**115.8**	117.14
MIDORI	79.9	74.5	**71.9**	71.9	71.9	71.9	74.56
PRINCE ${M_0}$, ${M_1}$	79.9	74.5	**71.9**	71.9	71.9	71.9	74.56
PRIDE ${L_0}$, ${L_3}$	79.9	74.5	**71.9**	71.9	71.9	71.9	74.56
QARMA128 (Avanzi, 2017)	159.8	–	145.8	145.8	**144.5**	144.5	149.12
QARMA64	79.9	74.5	**71.9**	71.9	71.9	71.9	74.56
SMALLSCALE AES	143.1	**111.8**	123.8	123.8	121.8	118.4	115.82
TWOFISH (Schneier et al., 1998)	369.6	317.5	338.9	312.9	306.9	**293.5**	**293.53**

**Note:**

Bold values indicate the best results.

**Table 7:** The global optimization result of $G{H_1}$ with 19 XORs, depth 3, and 101.84 GE in ASIC4 by using the depth-bounded version of BDKCI.

No	Operation	No	Operation
1	${y_1} = {x_1} \oplus {x_6} \oplus {x_8} \oplus {x_{12}}$	11	${y_6} = {x_1} \oplus {y_1} \oplus {y_8} \oplus {y_{12}}$
2	${y_{12}} = {x_2} \oplus {x_5} \oplus {x_{10}} \oplus {x_{12}}$	12	${t_{11}} = {x_0} \oplus {x_6} \oplus {x_{12}} \oplus {x_{13}}$
3	${y_0} = {x_0} \oplus {x_5} \oplus {x_{11}} \oplus {x_{15}}$	13	${y_{13}} = {y_0} \oplus {y_8} \oplus {t_{11}}$
4	${y_{11}} = {x_2} \oplus {x_4} \oplus {x_{11}} \oplus {x_{14}}$	14	${y_9} = {x_1} \oplus {x_9} \oplus {t_{11}}$
5	${y_{15}} = {x_1} \oplus {x_4} \oplus {x_9} \oplus {x_{15}}$	15	${y_4} = {x_4} \oplus {y_1} \oplus {t_{11}}$
6	${y_5} = {x_0} \oplus {y_0} \oplus {y_{11}} \oplus {y_{15}}$	16	${t_{15}} = {x_7} \oplus {x_9} \oplus {x_{14}} \oplus {x_{13}}$
7	${t_6} = {x_3} \oplus {x_5} \oplus {x_{12}} \oplus {x_{15}}$	17	${y_2} = {x_{11}} \oplus {y_{11}} \oplus {t_{15}}$
8	${y_3} = {y_{11}} \oplus {y_{12}} \oplus {t_6}$	18	${y_{10}} = {x_{12}} \oplus {y_2} \oplus {y_5} \oplus {y_{12}}$
9	${y_7} = {x_7} \oplus {y_0} \oplus {t_6}$	19	${y_{14}} = {y_4} \oplus {t_{15}}$
10	${y_8} = {x_0} \oplus {x_8} \oplus {t_6}$	–	

**Table 8:** The global optimization results of 4 × 4 involutory and MDS matrices over ${{\bf{F}}_{{2^4}}}$ by using the depth-bounded version of BDKCI.

Ref.	Type	#XOR2	#XOR3	#XOR4	GC	ASIC1 (GE)	ASIC2 (GE)	ASIC3 (GE)	ASIC4 (GE)	Depth
Sim et al. (2015)	Hadamard, Involutory	–	–	20	20	100	110	125	119.8	3
Li & Wang (2016)	Hadamard, Involutory	–	–	20	20	100	110	125	119.8	3
Sarkar & Syed (2016)	Involutory	2	5	12	19	80.25	88.537	101	**101.84**	3
Jean et al. (2017)	Involutory	1	4	15	20	90	99.341	113.05	111.82	4
$G{H_1}$	GHadamard, Involutory	1	7	11	19	**79.75**	**88.486**	**100.65**	**101.84**	3

**Note:**

Bold values indicate the best results.

## Conclusion and future works

In this article, we give a new heuristic SBP to search for efficient circuit implementations of a given linear layer. By considering low-latency criteria, our heuristic performs better results under the minimum circuit depth metric for $16 \times 16$ binary matrices compared to various global optimization algorithms. In this respect, especially by considering low-latency and low-cost circuits of $4 \times 4$ involutory MDS matrices over ${{\bf{F}}_{{2^4}}}$, we give a new lightest record, which can be implemented by only 41 g-XORs with depth 3. Additionally, in order to further optimize the results, we incorporate a circuit depth limit into the BDKCI algorithm. The proposed depth-bounded version of BDKCI has allowed us to achieve even better results. Above all, we give a circuit of AES MixColumn with 240.95 GE in ASIC4 library, which is the best result achieved thus far. Much better, our new $4 \times 4$ involutory MDS matrix requires 79.75, 88.486, 100.65, and 101.84 GE in the ASIC1, ASIC2, ASIC3, and ASIC4 libraries, respectively. That result is the lightest and superior to the state-of-the-art results. It should be noted that by conducting more runs of our depth-bounded version of BDKCI implementation, there is potential for further improvement of all these circuit results given in this article.

### Future works

Future research directions include optimizing SBP heuristic for larger matrices. Alternatively, it would be intriguing to explore the conversion of SBP into a multiple-input XOR gate version for improved results.

## Additional Information and Declarations

### Data Availability

The following information was supplied regarding data availability: The SBP (Superior Boyar-Peralta) repository are available at GitHub and Zenodo: - https://github.com/demirmehmet0/SBP. - demirmehmet0, & Meltem Kurt Pehlivanoglu. (2023). demirmehmet0/SBP: Initial Release (0.1.1). Zenodo. https://doi.org/10.5281/zenodo.10080462.
